# Feasibility and acceptability of a televideo physical activity and nutrition program for recent kidney transplant recipients

**DOI:** 10.1186/s40814-020-00672-4

**Published:** 2020-09-10

**Authors:** Cheryl A. Gibson, Aditi Gupta, J. Leon Greene, Jaehoon Lee, Rebecca R. Mount, Debra K. Sullivan

**Affiliations:** 1 Department of Internal Medicine, University of Kansas Medical Center, 3901 Rainbow Blvd., MS1020, Kansas City, KS 66160 USA; 2 Department of Internal Medicine, Division of Nephrology and Hypertension, Kidney Institute, University of Kansas Medical Center, Kansas City, KS USA; 3 Department of Health, Sports, and Exercise Sciences, University of Kansas, Lawrence, KS USA; 4 Department of Educational Psychology and Leadership, Texas Tech University, Lubbock, TX USA; 5 Department of Dietetics and Nutrition, University of Kansas Medical Center, Kansas City, KS USA

**Keywords:** Kidney transplant, Weight gain, Diet, Physical activity

## Abstract

**Background:**

Post-transplant weight gain affects 50–90% of kidney transplant recipients adversely affecting survival, quality of life, and risk for diabetes and cardiovascular disease. Diet modification and physical activity may help prevent post-transplant weight gain. Methods for effective implementation of these lifestyle modifications are needed. The objective of this study is to assess the feasibility and acceptability of a remotely delivered nutrition and physical activity intervention among kidney transplant recipients. Secondary aims were to estimate the effectiveness of the intervention in producing changes in physical activity, qualify of life, fruit and vegetable intake, and consumption of whole grains and water from baseline to 6 months.

**Methods:**

A randomized controlled study for stable kidney transplant recipients between 6 and 12 months post-transplantation was conducted. Participants were randomly assigned 1:1 to a technology-based, lifestyle modification program (intervention) or to enhanced usual care (control).

**Results:**

The first 10 kidney transplant recipients screened were eligible and randomized into the intervention and control groups with no significant between-group differences at baseline. Health coaching attendance (78%) and adherence to reporting healthy behaviors (86%) were high. All participants returned for final assessments. The weight in controls remained stable, while the intervention arm showed weight gain at 3 and 6 months. Improvements were found for physical activity, quality of life, and fruit and vegetable intake in both groups. All participants would recommend the program to other transplant recipients.

**Conclusions:**

Our data suggest that a remotely delivered televideo nutrition and physical activity intervention is feasible and valued by patients. These findings will aid in the development of a larger, more prescriptive, randomized trial to address weight gain prevention.

**Trial registration:**

Clinicaltrials.gov Identifier NCT03697317. Retrospectively registered on October 5, 2018.

## Key messages regarding feasibility

- What uncertainties existed regarding the feasibility?
  There is no information on the safety, acceptability, and adherence of a nutrition and physical activity program delivered by televideo among kidney transplant patients to help prevent post-transplant weight gain.
- What are the key feasibility findings?
  The nutrition and physical activity program was safe with good attendance and acceptance among the kidney transplant patients.
- What are the implications of the feasibility findings for the design of the main study?
  The results of this feasibility study can inform the design, help calculate an adequate sample size, and determine outcome measures to enhance the development of a randomized controlled trial.

## Background

The number of kidney transplant recipients (KTR) has rapidly increased to greater than 200,000 living recipients, more than doubling in the last 17 years alone [1]. Post-transplant weight gain is common, with KTR gaining an average of 10–35% of their body weight [2–8]. This weight gain is multifactorial and can be associated with a development of metabolic syndrome, new-onset diabetes after transplantation, increased risk of cardiovascular disease, and/or allograft loss [9–13].

Because dietary patterns and physical activity (PA) play a major role in weight maintenance and disease, nutritious diets and maintenance of a healthy weight after transplantation is recommended for KTR [3, 14, 15]. Due to a limited research base, there are no specific nutrition and PA guidelines for KTR; nonetheless, KTR should adhere to the same nutrition and PA guidelines as the general population. General healthy guidelines include limiting the consumption of processed and red meats, eating a plant-based diet, choosing whole grains over refined grains, drinking water in place of sugar-sweetened beverages, and maintaining a healthy weight. Higher levels of PA among KTR have been linked to a reduced risk of cardiovascular disease, weight gain, and diabetes [16–18].

Weight gain affects 50–90% of KTR [19] and is recognized as a common problem for patients [20]. However, few interventions that target healthy lifestyle behaviors to address post-transplantation weight gain have been undertaken. This pilot study aimed to fill the research gap through the development of a nutrition and PA protocol to increase healthy lifestyle behaviors in KTR. Utilizing remote delivery of video conferencing technology allows KTR to interact with health coaches and other KTR for support and encouragement in achieving a healthy lifestyle.

Our specific aim was to conduct formative research to test the feasibility of our intervention post-transplantation. As part of this aim, we assessed participant recruitment (achievement of proposed sample size), attendance (per weekly online sessions/assessment visits), and adherence (number of participants at 3 months and 6 months who were reporting data). Our secondary aims were to estimate the effectiveness of the intervention program in producing changes in PA, fruit and vegetable intake, whole-grain consumption, and water consumption. We also tracked weight gain from baseline to 6 months. By demonstrating the feasibility and efficacy of an in-home televideo health coaching program, this intervention can fill an important gap in the literature and lead to evidence-based data to guide clinical management.

## Methods

This trial was approved by the University of Kansas Medical Center Institutional Review Board (Reviewing IRB: IRB00000161; IRB# STUDY00140695) and has been registered at clinicaltrials.gov (Identifier NCT03697317). All participants provided written informed consent before data collection.

Our study design draws from the Social Cognitive Theory (SCT) of behavioral change [21]. SCT explains behavior as a triadic relationship between personal, behavioral, and environmental factors. The constructs targeted and operationalized in this intervention include self-efficacy, outcome expectations, self-monitoring, goal setting, perceived facilitators and barriers to changes, role modeling, and environmental factors.

We conducted a single-blind randomized controlled trial to assess the feasibility of conducting a fully powered effectiveness trial. Participants were randomly assigned 1:1 to a technology-based, interactive, and tailored lifestyle modification program (TLC4KTx; intervention) or to enhanced usual care (eUC; control). The randomization schedule was generated using SAS 9.4 (SAS Institute, 2002-2012). Investigators recruiting participants to the trial did not have access to the randomization scheme and remained blinded until primary analyses were complete. Additionally, the randomization code was computer generated, and the research team member randomizing participants did not know the allocation sequence in advance.

**Fig. 1:**
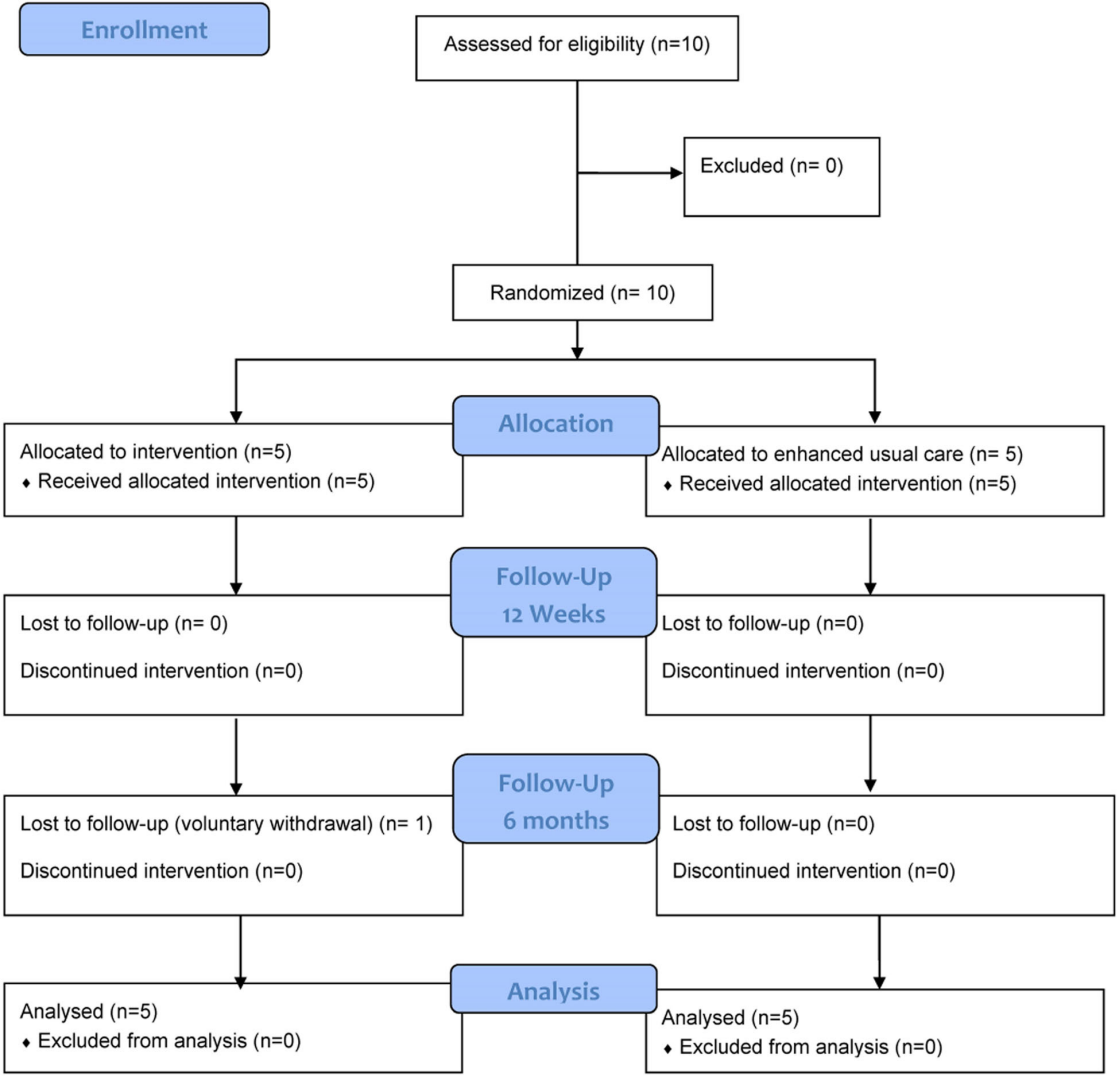
Study flow diagram

The program duration was 6 months with 12 weeks of weekly 1-h health coaching delivered remotely followed by 12 weeks of maintaining healthy behaviors (Fig. 1). Additionally, we collected qualitative feedback from both intervention and control participants to understand their experiences related to study participation and assess facilitators and barriers to participation in diet and PA behaviors.

### Sample size and power

Our goal for this pilot feasibility study was to obtain unbiased effect size estimates for TLC4KTx as evidence of premise. We did not anticipate adequate power with our sample size. We calculated minimum detectable effect size to provide an insight for the smallest “true” effect for which this study could find statistical significance with 80% chance [22]. When a correlation of 0.60 was assumed among repeated measurements, minimum detectable effect size was *f* = 0.39 indicating that this study could achieve ≥ 80% power if the true effect of TLC4KTx is large.

### Recruitment

We recruited kidney transplant recipients between 6 and 12 months from their transplant surgery from the transplant clinic of an academic medical center. Since the initial months post-transplant are critical for the long-term outcome of the allograft, we waited 6 months after the surgery in order to avoid additional instructions and tasks during this critical period to allow full recovery from surgery. We chose 12 months as the cut-off for enrollment since per our transplant center policy, after the first year, the patients return to their general nephrologists for their care. Inclusion criteria included (1) age 18 years or older at the time of transplant; (2) kidney allograft recipient; (3) with functioning allograft (not on dialysis); (4) BMI > 22 kg/m^2^; (5) availability to participate in assessments over 6 months; (6) ability to speak and understand English; (7) ability to report data weekly by at least one of three alternative methods: telephone, email, or fax; and (8) access to wireless Internet.

Exclusion criteria included (1) multi-organ transplant recipient; (2) uncontrolled diabetes with hemoglobin A1c ≥ 8%; (3) pregnancy; (4) participation in a formal weight management, nutrition, or PA program; (5) diagnosis of a psychiatric illness such as major depressive episodes, schizophrenia, or bipolar disorder; (6) dietary restrictions, such as vegetarianism or severe food allergies; (7) inability to perform moderate to vigorous PA; (8) unwillingness to be randomized; and (9) receiving supplemental nutrition (i.e., total parenteral nutrition, nasogastric tube feedings). Informed consent was obtained from all individual participants included in the study.

### Technology

Participants received a tablet computer and a fitness tracker for study-related use, regardless of group assignment. Device use entailed attending remotely delivered televideo health coaching sessions and required weekly reporting of healthy lifestyle behaviors.

### Healthy lifestyle calendars

**Fig. 2:**
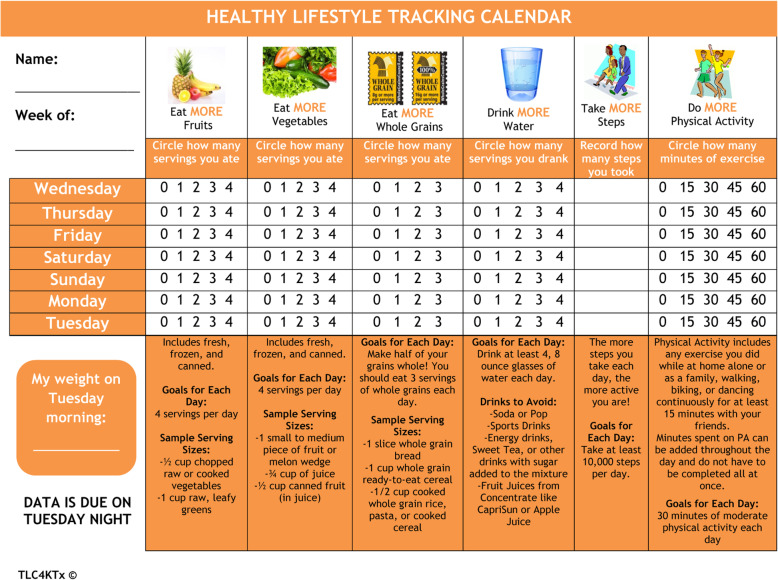
Healthy lifestyle tracking calendar

All participants tracked their healthy lifestyle behaviors and reported those to the study team on a weekly basis. Healthy lifestyle calendars were provided to participants on the tablet computers to complete each week (Fig. 2). Healthy behaviors to track daily included consumption of fruit, vegetable, and whole grain servings; number of steps taken; and minutes of PA achieved.

### Lifestyle modification group (TLC4KTx)

Individuals randomized to this group participated in weekly 1-h health coaching sessions remotely delivered by video conferencing for 12 weeks. These sessions, led by a registered dietitian and adaptive PA expert, taught individuals about nutrition, PA, and behavioral strategies tailored to KTR. Remotely delivered coaching sessions were split between interactive discussion and group PA. During the discussions, individuals were taught appropriate portion sizes and healthy cooking skills, and sample menus were shared. Specific issues related to KTR, such as exercise capacity, nutrient needs, and health-related quality of life, were addressed. The goal of the PA portion of the intervention was for individuals to participate in three, 10–15-min bouts of moderate intensity PA (i.e., 3–6 METs) daily in the home settings. Participants were instructed to accumulate at least 150 min of moderate intensity PA per week, as tolerated. This approach satisfies the American College of Sports Medicine, the 2008 Physical Activity Guidelines for Americans, and Centers for Disease Control guidelines for PA and provides PA in a context other than competitive sports [23, 24].

### Enhanced usual care (eUC)

Participants randomized to the eUC group were provided standard recommendations to eat a well-balanced diet and perform PA as tolerated. The eUC group completed weekly healthy lifestyle tracking calendars but did not attend weekly health coaching or PA classes. Educational materials were accessible on the tablet computers, and participants could review the materials at any time.

### Nutrition education content

Health coaching session content and written education materials were developed using healthy eating goals for individuals using the Dietary Guidelines and MyPlate goals adapted for a modified Dietary Approaches to Stop Hypertension (DASH) dietary pattern [25]. The DASH dietary pattern involves an emphasis on the consumption of fruits, vegetables, whole grains, and low-fat dairy. While the DASH dietary pattern was originally developed and studied for hypertension, it has been utilized often for weight management. A recent systematic review indicated that adults lose more weight on the DASH diet compared to controls [26]. Although the DASH dietary pattern has not been studied exclusively in this population, it is appropriate due to the prevalence of cardiovascular disease risk factors among this population.

### Assessments

Measures included sociodemographic information, relevant medical history, medication use, anthropometrics (height, weight, waist circumference), blood pressure, PA measures including accelerometry, quality of life measures, and dietary intake using 3-day food records. Assessments took place at baseline, week 12, and end of study (week 24).

#### Anthropometric measurements

Duplicate measures of weight to the nearest 0.1 kg using a calibrated digital scale (Model #PS6600, Belfour, Saukville, WI) were taken without shoes, socks, and heavy clothing. Height was measured in duplicate with a portable stadiometer (Model #IP0955, Invicta Plastics Limited, Leicester, UK). Body mass index (BMI) was calculated as weight (kg)/height (m^2^). Waist circumference, serving as a surrogate for abdominal adiposity, was measured three times with the average of the two closest values recorded as the outcome variable [27].

#### Blood pressure

Resting systolic blood pressure (SBP) and diastolic blood pressure (DBP) was determined by the average of two measurements taken within a 5-min interval after participants had rested for at least 5 min. Blood pressure was measured using an automated procedure.

#### Physical activity

Participants wore an ActiGraph Model GT3X+ accelerometer (ActiGraph LLC, Pensacola, FL) for 7 consecutive days on a belt over the non-dominant hip during all waking hours. The outcome variables included the average movement counts per minute and the average minutes per day spent in moderate and vigorous PA over a 7-day period using age specific cut-points.

#### Quality of life

To assess QoL, a kidney disease-specific questionnaire comprised of 25 questions in 5 dimensions (physical symptoms, fatigue, uncertainty/fear, appearance, and emotions) was administered. Intraclass correlation coefficients (ICC) indicate the questionnaire is reproducible in stable renal transplant patients (ICC between 0.82 and 0.91) and responsive to change [28].

#### Dietary intake

Total dietary intake was determined using 3-day food records (2 weekdays and 1 weekend day). Food record data was entered into the Nutrient Data System for Research (NDS-R version 2017) for energy, nutrient, and food group analyses. Using the 2010 Healthy Eating Index (HEI-2010), as described by Guenther et al. [20], diet quality was assessed. A total HEI-2010 score was calculated as a sum of 12 subscale scores, with a higher total score indicative of a better diet quality (maximum score of 100). Current medical status, medications, and treatment plan were obtained from the transplant team and verified with the participant.

#### Qualitative interviews

At the end of the study, all participants were invited to take part in a semi-structured telephone interview conducted by an experienced, qualitative researcher with whom they had no previous contact. The interviews allowed participants to share their experiences with following the prescribed PA and diet regimen in more depth to augment the findings. The interviews were conducted using an interview guide developed by study team members. All interviews were digitally recorded and transcribed verbatim.

### Statistical analysis

Descriptive statistics were utilized to evaluate the feasibility of TLC4KTx. Sample demographics were also compared between the intervention and eUC groups to determine success/failure of the group randomization executed, using independent-samples *t* test (with Satterthwaite approximation if necessary) and chi-square (or Fisher’s exact test, as appropriate). An effect size, Cohen’s *d* or Cramér’s *V*, was calculated for each comparison.

To evaluate the efficacy of the intervention program, mixed modeling was conducted separately for the anthropometric, QoL, PA, and diet quality measures. Models estimated overall group difference across time (group effect), change over time (time effect), and group difference in this change (group-by-time interaction), while accounting for the clustering of measurements (level 1) repeated for participants (level 2). Models also accounted for key demographic variables such as age, sex, race, and education level, thereby providing unbiased estimates of the intervention effects. A proper error covariance structure was determined for each outcome variable by evaluating relative model fit (e.g., Akaike information criterion, adjusted Bayesian information criterion). Full information maximum likelihood (FIML) was employed for model estimation. All quantitative analyses were conducted using SAS 9.4 (SAS Institute, 2002–2012).

Thematic analysis was performed using the transcripts of the semi-structured interviews to elucidate participant views of the strengths and weaknesses of the intervention components, what might make adherence to the intervention components easier, and the participants’ recommendations on how to improve the intervention. Team members reviewed the transcripts several times, coded and grouped common themes across interviews, and compared and discussed themes until consensus was achieved [29]. Specific quotes were selected to represent themes that emerged from the transcripts and captured the sentiment of other participants.

## Results

### Participants

Screening and enrollment occurred from 17 August 2017 to 27 September 2017. Ten patients enrolled in the study; 5 were randomized to the intervention group and 5 to the control group. One person withdrew after 3 months because of jury duty and a work schedule conflict. Table 1 displays the baseline characteristics of the participants. There were equal numbers of male (50%) and female (50%) participants with an average age of 44.60 ± 10.02 years. Table 2 provides key kidney transplant characteristics of the study population.

**Table 1:** Baseline characteristics of the study participants

	Total (*n* = 10)	Control (*n* = 5)	Intervention (*n* = 5)	*p* value	*V/d*
**Gender**				1.00	0.2
Male	5	2	3		
Female	5	3	2		
**Race**				1.00	0.3
Hispanic or Latino	1	1	0		
Non-Hispanic White	5	2	3		
Black or African	2	1	1		
Multi-racial	2	1	1		
**Age in years**	44.6 ± 10.0	44.0 ± 11.0	45.2 ± 10.2	0.9	0.1
**Education**				1.00	0.3
High school graduate	4	2	2		
Some college	5	3	2		
College graduate	1	0	1

**Table 2:** Kidney transplant characteristics

	Total	Control	Intervention
**Reason for transplant**			
Chronic kidney disease	2	1	1
End stage renal disease	3	1	2
Polycystic kidney disease	1	1	0
Other	4	2	2
**Median duration of dialysis (months)**		11.4	32.7
**Type of transplant donor**			
Live	4	2	2
Deceased	6	3	3
**Immunosuppressive medication**			
Tacrolimus	10	5	5
Prednisone	8	3	5
Mycophenolic acid (Myfortic)	10	5	5

### Feasibility of the program

The intervention and control groups did not differ in terms of age (*p* = 0.86, *d* = 0.11), gender (*p* = 1.00, *V* = 0.20), race (*p* = 1.00, *V* = 0.35), and education level (*p* = 1.00, *V* = 0.35). The attendance rate of health coaching sessions was 78% for the 12 sessions. Absences were due to illness or conflicts with school or work schedules. Adherence to reporting healthy behaviors was 86%. Technological issues were the main barriers to full data reporting. All 10 participants returned for week 12 study assessments.

### Efficacy of the program

Table 3 provides descriptive statistics of the anthropometric, QoL, PA, and diet quality measures separately for the intervention and control groups as well as the results of group comparisons.

**Table 3:** Descriptive statistics of study variables

Variable	Total (*N* = 10)		Control (*n* = 5)		Intervention (*n* = 5)			
	*N*	*M* ± SD	*n*	*M* ± SD	*n*	*M* ± SD	*p*	*d*
**Weight (kg)**								
0 month	10	94.14 ± 21.13	5	90.24 ± 20.83	5	98.04 ± 23.09	0.590	0.159
3 months	10	95.69 ± 21.00	5	90.64 ± 19.03	5	100.74 ± 23.80	0.480	0.210
6 months	9	96.67 ± 21.17	5	90.32 ± 17.91	4	104.60 ± 24.84	0.376	0.321
**BMI (kg/m**^**2**^**)**								
0 month	10	32.67 ± 5.64	5	31.51 ± 7.93	5	33.83 ± 2.31	0.559	0.178
3 months	10	33.11 ± 5.79	5	31.62 ± 7.87	5	34.59 ± 2.86	0.450	0.225
6 months	9	33.77 ± 5.94	5	31.73 ± 7.40	4	36.31 ± 2.37	0.250	0.355
**Waist circumference (cm)**								
0 month	10	105.17 ± 18.56	5	101.45 ± 23.07	5	108.89 ± 14.43	0.558	0.173
3 months	10	105.27 ± 15.82	5	101.55 ± 19.54	5	108.98 ± 12.13	0.491	0.204
6 months	9	105.38 ± 16.73	5	100.81 ± 19.22	4	111.09 ± 13.27	0.376	0.279
**Systolic blood pressure (mmHg)**								
0 month	10	126.10 ± 7.26	5	124.80 ± 7.92	5	127.40 ± 7.20	0.602	0.154
3 months	10	131.20 ± 11.16	5	130.60 ± 7.92	5	131.80 ± 14.72	0.878	0.045
6 months	9	129.78 ± 13.90	5	122.60 ± 10.50	4	138.75 ± 13.23	0.096	0.651
**Diastolic blood pressure (mmHg)**								
0 month	10	75.80 ± 10.49	5	71.00 ± 8.66	5	80.60 ± 10.71	0.158	0.441
3 months	10	77.50 ± 10.44	5	72.20 ± 4.55	5	82.80 ± 12.42	0.111	0.507
6 months	9	80.00 ± 10.84	5	73.80 ± 7.85	4	87.75 ± 9.32	0.054	0.774
**Counts/min**								
0 month	61	275.69 ± 173.48	32	349.14 ± 201.84	29	194.63 ± 80.09	0.000	0.176
3 months	33	279.17 ± 120.40	21	303.72 ± 132.58	12	236.21 ± 83.93	0.123	0.130
6 months	38	251.98 ± 143.57	19	321.76 ± 166.19	19	182.21 ± 66.88	0.002	0.253
**Moderate PA min/day**								
0 month	57	26.04 ± 25.51	31	34.68 ± 30.44	26	15.73 ± 11.97	0.003	0.144
3 months	29	20.69 ± 20.65	18	27.83 ± 22.08	11	9.00 ± 11.08	0.005	0.243
6 months	36	20.14 ± 23.81	19	30.68 ± 28.62	17	8.35 ± 6.26	0.004	0.241
**Vigorous PA min/day**								
0 month	2	3.00 ± 2.83	2	3.00 ± 2.83	0	–	–	–
3 months	4	10.25 ± 8.54	3	7.67 ± 8.33	1	18.00 ± 0.00	–	–
6 months	3	5.67 ± 8.08	3	5.67 ± 8.08	0	–	–	–
**KTQ: physical symptoms**								
0 month	10	3.25 ± 0.82	5	3.37 ± 1.13	5	3.13 ± 0.45	0.679	0.122
3 months	10	4.97 ± 0.95	5	5.53 ± 1.06	5	4.40 ± 0.28	0.075	0.652
6 months	9	4.70 ± 1.34	5	5.47 ± 0.43	4	3.75 ± 1.53	0.107	0.807
**KTQ: fatigue**								
0 month	10	4.00 ± 1.40	5	4.00 ± 1.94	5	4.00 ± 0.80	1.000	0.000
3 months	10	5.28 ± 0.94	5	5.92 ± 0.98	5	4.64 ± 0.17	0.042	0.818
6 months	9	4.93 ± 1.41	5	5.72 ± 0.91	4	3.95 ± 1.37	0.077	0.749
**KTQ: uncertainty/fear**								
0 month	10	4.13 ± 0.85	5	4.30 ± 1.10	5	3.95 ± 0.60	0.548	0.177
3 months	10	4.90 ± 1.20	5	5.70 ± 0.82	5	4.10 ± 0.99	0.024	0.786
6 months	9	4.75 ± 1.62	5	5.60 ± 1.15	4	3.69 ± 1.59	0.095	0.671
**KTQ: appearance**								
0 month	10	5.35 ± 1.42	5	6.15 ± 1.14	5	4.55 ± 1.27	0.069	0.594
3 months	10	5.60 ± 1.17	5	6.55 ± 0.41	5	4.65 ± 0.82	0.002	1.308
6 months	9	5.39 ± 1.47	5	6.55 ± 0.45	4	3.94 ± 0.66	0.001	2.267
**KTQ: emotions**								
0 month	10	4.50 ± 1.05	5	4.60 ± 1.09	5	4.40 ± 1.12	0.782	0.081
3 months	10	5.10 ± 1.11	5	5.60 ± 1.22	5	4.60 ± 0.82	0.166	0.431
6 months	9	4.78 ± 1.64	5	5.87 ± 1.39	4	3.42 ± 1.39	0.029	0.824
**HEI: total fruit**								
0 month	10	1.80 ± 1.32	5	2.60 ± 1.14	5	1.00 ± 1.00	0.046	0.667
3 months	9	2.89 ± 1.96	4	2.75 ± 1.71	5	3.00 ± 2.35	0.864	0.055
6 months	9	3.11 ± 1.83	5	2.80 ± 1.48	4	3.50 ± 2.38	0.604	0.175
**HEI: whole fruit**								
0 month	10	2.60 ± 1.90	5	3.40 ± 1.82	5	1.80 ± 1.79	0.198	0.397
3 months	9	2.89 ± 2.32	4	2.50 ± 2.38	5	3.20 ± 2.49	0.682	0.134
6 months	9	3.67 ± 1.80	5	3.60 ± 1.34	4	3.75 ± 2.50	0.911	0.038
**HEI: total vegetable**								
0 month	10	3.20 ± 1.32	5	3.60 ± 1.67	5	2.80 ± 0.84	0.367	0.270
3 months	9	3.67 ± 1.12	4	4.50 ± 1.00	5	3.00 ± 0.71	0.033	0.846
6 months	9	4.11 ± 0.93	5	4.40 ± 0.89	4	3.75 ± 0.96	0.328	0.331
**HEI: green and bean**								
0 month	10	2.40 ± 2.22	5	3.00 ± 2.35	5	1.80 ± 2.17	0.425	0.238
3 months	9	1.78 ± 2.22	4	2.75 ± 2.63	5	1.00 ± 1.73	0.267	0.387
6 months	9	2.56 ± 2.19	5	2.40 ± 2.51	4	2.75 ± 2.06	0.829	0.070
**HEI: whole grain**								
0 month	10	4.20 ± 2.74	5	2.60 ± 1.34	5	5.80 ± 2.95	0.058	0.625
3 months	9	7.22 ± 3.42	4	9.00 ± 1.41	5	5.80 ± 4.02	0.177	0.454
6 months	9	4.78 ± 2.68	5	4.40 ± 3.21	4	5.25 ± 2.22	0.668	0.138
**HEI: dairy**								
0 month	10	4.70 ± 2.67	5	3.80 ± 3.42	5	5.60 ± 1.52	0.313	0.304
3 months	9	4.67 ± 2.29	4	4.25 ± 2.50	5	5.00 ± 2.35	0.657	0.146
6 months	9	4.78 ± 2.33	5	4.00 ± 2.00	4	5.75 ± 2.63	0.292	0.363
**HEI: total protein**								
0 month	10	4.50 ± 0.71	5	4.40 ± 0.89	5	4.60 ± 0.55	0.681	0.121
3 months	9	4.33 ± 0.71	4	4.25 ± 0.96	5	4.40 ± 0.55	0.775	0.096
6 months	9	4.44 ± 0.73	5	4.60 ± 0.55	4	4.25 ± 0.96	0.510	0.225
**HEI: sea plant protein**								
0 month	10	1.90 ± 2.02	5	1.00 ± 1.41	5	2.80 ± 2.28	0.172	0.424
3 months	9	1.78 ± 1.56	4	1.50 ± 1.29	5	2.00 ± 1.87	0.665	0.139
6 months	9	1.89 ± 2.09	5	1.60 ± 1.82	4	2.25 ± 2.63	0.673	0.141
**HEI: fatty acid**								
0 month	10	5.20 ± 2.44	5	4.20 ± 1.30	5	6.20 ± 3.03	0.213	0.383
3 months	9	4.78 ± 2.68	4	6.50 ± 2.65	5	3.40 ± 1.95	0.082	0.648
6 months	9	6.22 ± 3.23	5	7.60 ± 2.30	4	4.50 ± 3.70	0.165	0.499
**HEI: refined grain**								
0 month	10	4.20 ± 3.71	5	4.00 ± 3.87	5	4.40 ± 3.97	0.876	0.046
3 months	9	6.22 ± 3.73	4	8.00 ± 1.41	5	4.80 ± 4.55	0.222	0.405
6 months	9	6.00 ± 3.28	5	6.40 ± 2.51	4	5.50 ± 4.43	0.710	0.125
**HEI: sodium**								
0 month	10	3.10 ± 3.54	5	3.20 ± 2.86	5	3.00 ± 4.47	0.935	0.024
3 months	9	2.78 ± 2.59	4	3.75 ± 3.30	5	2.00 ± 1.87	0.346	0.327
6 months	9	3.22 ± 1.99	5	3.00 ± 2.55	4	3.50 ± 1.29	0.734	0.108
**HEI: empty kcal**								
0 month	10	15.50 ± 2.68	5	15.40 ± 2.61	5	15.60 ± 3.05	0.914	0.032
3 months	9	15.89 ± 3.30	4	18.25 ± 2.06	5	14.00 ± 2.92	0.044	0.756
6 months	9	15.89 ± 3.33	5	16.80 ± 2.17	4	14.75 ± 4.50	0.395	0.295
**HEI: total**								
0 month	10	53.30 ± 14.15	5	51.20 ± 8.07	5	55.40 ± 19.35	0.666	0.127
3 months	9	58.89 ± 11.32	4	68.00 ± 6.83	5	51.60 ± 8.47	0.017	0.971
6 months	9	60.67 ± 12.11	5	61.60 ± 5.32	4	59.50 ± 18.72	0.840	0.080

*KTQ* Kidney Transplant Questionnaire, *HEI* Healthy Eating Index

#### Anthropometrics

The weight change from baseline to 6 months was 5.50 ± 2.16 kg (+ 6.7%) for intervention participants compared to 0.08 ± 4.12 kg (< 1%) for controls (*p* = 0.04, *d* = 0.72). The group-by-time interaction was also significant in the mixed modeling (*p* = 0.003) indicating that after controlling for participants’ age, sex, race, and education level (covariates), weight gain was significantly greater in the intervention group compared to the control group. Similarly, the intervention group had greater gain in BMI over the 6-month period either without (2.19 ± 1.16 vs. 0.22 ± 1.92 kg/m^2^, *p* = 0.10, *d* = 0.55) or with controlling for the covariates (group-by-time interaction *p* = 0.03). The intervention and control groups did not show a significant difference in waist circumference in the 0–6-month change either before (3.35 ± 4.28 vs. 0.64 ± 5.55 cm, *p* = 0.26, *d* = 0.25) or after controlling for the covariates (group-by-time interaction *p* = 0.20). However, the increase in waist circumference between 3 and 6 months was significantly greater for intervention participants (2.61 ± 2.12 vs. 0.74 ± 1.51 cm, *p* = 0.04, *d* = 0.50).

#### Blood pressure

The changes in both SBP and DBP did not differ between the control and intervention groups (see Table 3; all *p* > 0.05). Consistent with this result, neither the group effect (*p* = 0.56 and 0.06, respectively) nor the group-by-time interaction (*p* = 0.24 and 0.30, respectively) was significant in the mixed modeling that accounted for the covariates.

#### Physical activity

For intervention participants, the average movement counts per minute increased during the first 3 months of the intervention (194.63 ± 80.09 to 236.21 ± 83.93) but decreased at 6 months to below the baseline level (182.21 ± 66.88). Similar results were found for control participants—i.e., increase in the counts at 3 months but a decrease by 6 months (see Table 3). Correspondingly, neither the group effect (*p* = 0.29) nor the group-by-time interaction (*p* = 0.80) was significant in the mixed modeling. The average minutes per day spent in moderate PA decreased over the 6-month period in both the intervention and control groups (see Table 3). Because of the small number of observations, reliable results were not obtained in the case of vigorous PA.

#### Quality of life

Intervention participants showed an improvement in every domain of their QoL (see Table 3). Particularly, the improvements in physical symptoms during the first 3 months of the intervention (1.27 ± 0.62, *p* = 0.01, *d* = 2.05) and emotions between 0 and 6 months (0.79 ± 0.41, *p* = 0.03, *d* = 1.91) were statistically significant. For control participants, QoL was also enhanced in all domains. The change from baseline was significant for physical symptoms at 3 months (2.17 ± 1.09, *p* = 0.01, *d* = 1.98) and 6 months (2.10 ± 1.41, *p* = 0.03, *d* = 1.49); for fatigue at 3 months (1.92 ± 1.38, *p* = 0.04, *d* = 1.39) and 6 months (1.72 ± 1.12, *p* = 0.03, *d* = 1.54); for uncertainty/fear at 3 months (1.40 ± 0.98, *p* = 0.03, *d* = 1.43); and for emotions at 3 months (1.00 ± 0.57, *p* = 0.02, *d* = 1.77) and 6 months (1.26 ± 0.55, *p* = 0.01, *d* = 2.31). Overall, the improvements in QoL were greater for the control group compared to the intervention group and significantly in the fatigue and emotion domains (group-by-time interaction *p* = 0.04 and *p* = 0.001, respectively). The changes in the appearance domain did not differ between the control and intervention groups (all *p* > 0.05) either before (0.38 ± 1.13 vs. 0.40 ± 1.13, *p* = 0.34, *d* = 0.10) or after controlling for the covariates (group-by-time interaction *p* = 0.27).

### Dietary intake

In general, diet quality was improved over the 6-month period for both groups (see Table 3). Specifically, the HEI score change from baseline to 6 months in the intervention group was 2.25 ± 3.10 for total fruit intake; 1.50 ± 3.87 for whole fruit intake; and 0.75 ± 0.50 for total vegetable intake, which were higher (but not significantly higher) than the changes observed in the control group (0.20 ± 1.64, 0.20 ± 1.48, and 0.80 ± 0.84, respectively). Consistent with this result, the time effect was significant for the total fruit (*p* = 0.02) and vegetable intakes (*p* = 0.04) in the mixed modeling when participants’ age, sex, race, and education level were controlled.

#### Semi-structured interviews

The themes and the quotes represented below convey the most salient features identified by the participants about the study.

##### Theme 1: Strengths of the intervention components

Participants reported several strengths of the study and how it positively influenced their PA and eating behaviors.

*“…I liked it all because it [study], you know, it helped me think about the food you should be eating, like the vegetables every day and the fruit. It got me thinking about and eating a lot more than I really did....and exercising a bit more.”* (P4 Female)

*“…it got me off of my duff to be motivated to do it [physical activity] even more. And once I started doing it and I realized it was really helping me feel a lot better. It really gave me drive to continue to do it. It made me watch what I ate a lot closer than I ever have in my life.”* (P2 Male)

*“...It got me back to a place where I was consistently working out and then setting goals and attaining those goals. …visually seeing what I was eating and where I needed to fill in the gaps.”* (P7 Female)

*“I liked having access to the resources and the tools. Like I had questions and it was nice to have them answered.”* (P10 Male)

One participant stated that the study not only helped him improve his nutrition but also helped his family understand what to eat for better health.

*“It actually taught me what I should be eating and how I should be eating it. What is good for not only me but my family and the serving sizes…it actually has given me a new way of thinking and a way of doing things.”* (P5 Male)

##### Theme 2: Challenges brought about by the study components

Participants reported how they struggled with the reporting aspects of the study, especially completing the tracking calendar in a timely manner.

*“...having to keep up with the actual records, the actual written records.”* (P3 Male)

*“Well, just the keeping track of what you’re eating every day part of it was difficult for me because I’m busy and on the road a lot so it’s kind of difficult to keep track.”* (P7 Female)

Two participants indicated the way in which the data was shared was not optimal.

*“The way we had to record it. Trying to record it on that Dropbox thing.” (P8 Female)*

* "…like an easier way of submitting our trackers through like a website where we do may do like a multiple-choice type deal instead of us having to either print it or fax it...*" (P3 Male)

##### Theme 3: Adherence to study components

Although participants reported tracking was a challenge, they also indicated how keeping a daily tally increased accountability and developed a greater awareness of PA and dietary habits.

*“Made me watch what I ate a lot closer than I ever have in my life. That’s probably the main thing. I mean, I paid attention to what I was buying and what kind of calorie intake I was taking…it probably made me eat more fruit and vegetables than I have in my entire life.”* (P2 Male)

“*So it got me back to a place where I was consistently working out and then setting goals and attaining those goals…the least favorite part [tracking] is the most helpful part…I knew those things but it just made me accountable and cognizant of what I was actually doing and not doing versus what I thought I was doing.”* (P7 Female)

##### Theme 4: Improvements to the study components

Participants were asked whether they would recommend this program to other kidney transplant patients. All participants indicated they would recommend the program to others. In addition, when participants were asked about suggestions to improve the program, they offered a few recommendations.

One participant wanted access to the video conferencing session so that he could review how to perform the suggested exercises later.

*“I just think that needs to be available for the participants later on, not just for that time but…just so we can go back and see exactly what we’re doing and how to do it when we’re not together like that.”* (P5 Male)

Another participant wanted the study team to structure the study to avoid the holiday season.

*“I felt that what I would recommend is maybe do it at a different time, because I was easily defeated during the holidays. And the winter months. Felt defeated.”* (P9 Female)

Two participants wanted the study to last longer than the 3 months of weekly contact.

*“…like maybe the four months instead of just the three.”* (P10 Male)

*“…I just hate that it ended so soon. But it really did help me because you’re—after a transplant you’re pretty much stuck in the hospital so it is kind of a social thing, too. And it helps to see what other kidney transplant patients were going through…so it’s a really good support group without getting out of the house.”* (P9 Female)

## Discussion

The primary aim of this study was to determine whether a televideo intervention was feasible and acceptable to KTR. We found that the trained health coach and adaptive PA expert did not have difficulty in implementing the televideo health coaching program as designed. Our participants were enthusiastic about the study. All patients referred by our nephrologist consented for the study. The attendance rate for the health coaching sessions was reasonably high with illness and school or work conflicts accounting for missed sessions. The single-blind randomization yielded comparable groups in terms of baseline characteristics, which provides better insights (i.e., effect sizes) on sample size and power of statistical inference in a future, larger efficacy trial. Adherence to weekly reporting of healthy behaviors was also very good with adherence rates approaching 90%. Participants provided valuable feedback for designing future studies. Some reported struggling with the weekly reporting but indicated daily tracking helped to increase their awareness and accountability to healthy eating and PA goals. Only one participant did not complete the study because he was called to serve jury duty and accepted a new job that required an evening work schedule. Importantly, all study participants indicated they would recommend the program to other KTR, expressing how they valued the tailored information shared with them about healthy eating (i.e., nutrient needs, appropriate portion sizes, cooking skills, sample menus) and physical activities (e.g., brisk walking or jogging in place, repeated sit and stand movements, vertical jumps, lunges) that could be performed safely at home without the need for gym membership or specialized exercise equipment.

For our secondary aims, we wanted to estimate the effectiveness of the intervention program in producing changes in PA, QoL, fruit and vegetable intake, and consumption of whole grains and water from baseline to 6 months. Modest weight gain was noted from baseline to 3 months among both groups. Similarly, investigators of a randomized controlled study, involving intensive nutrition and exercise advice to prevent excessive weight gain among KTR, did not demonstrate any advantage over standard care in the first year after transplant although weight gain was relatively modest in both groups [30]. A non-randomized study found that significant weight gain can be attenuated with early intensive dietary advice and follow-up [31].

For our other secondary aims, the average number of minutes of PA increased significantly during the first 3 months but subsequently decreased over the 6-month period for both groups. Low daily PA and other lifestyle factors have been implicated in post-transplant weight gain [14, 32] suggesting the prevention of weight gain after transplantation involves tailored programs including PA and dietary interventions. Other researchers report dietary intake and PA show no significant relationship to weight gain at 6 months post-transplantation [19] and participation in a PA program alone does not prevent weight gain in the first post-transplantation year [33]. Among the general population, there is also insufficient evidence for the effectiveness of telehealth interventions that involve monitoring of physical activity, dietary intake, and eating habits and behavioral counseling for weight gain prevention [34].

Similar to other telehealth-delivered interventions targeting dietary patterns in adults with chronic diseases, we found improvement in diet quality and increases in fruit and vegetable intake [35]. As in other video contact studies, patients benefitted from weekly monitoring and behavioral prompting, facilitating greater adherence to dietary advice [36, 37]. In addition, the ability for patients to receive the intervention at home likely improved the rates of adherence and reduced attrition, offering significant advantages over face-to-face consultations and resulting in a high rate of patient acceptability [38].

In summary, our study provided a feasible and acceptable method of delivery for KT patients to participate in a nutrition and PA program but was not successful in preventing weight gain among the small sample of individuals. However, by building upon the positive aspects of this study, it can inform the future development of evidence-based programs tailored to KTR. Currently, there are limited data from randomized clinical trials, and well-designed intervention studies are needed to determine how best to prevent unnecessary weight gain after transplantation that can be translated into clinical practice.

## Lessons and limitations

The strength of this study is that it provides formative information on weight gain issues among recent KTR that can be used for a larger randomized controlled trial. Another key strength of this study was the use of technology to deliver the intervention, allowing for interactive sessions for nutrition education and PA sessions led by trained experts. Importantly, a significant limitation was that the weight loss estimates derived from this small study may not favor a larger study with statistically significant results. However, this was a feasibility study and not optimally designed to have power enough to detect conclusive differences between the intervention and control groups. Although the study was limited by its small sample size, the semi-structured interviews allowed for candid responses and augmented the findings. Larger sample sizes are needed in future studies to confirm the results of the study. Lastly, the study was conducted at one health center, limiting the generalizability of the findings.

## Conclusions

In this study, we wanted to determine whether our intervention was feasible and acceptable to KTR. Although the intervention group had greater weight gain than the control group, we found the televideo health coaching program to be a feasible and acceptable method of delivery for nutrition education and home-based PA among KT patients. With known adverse effects of post-transplant weight gain on morbidity and mortality, additional studies are needed to help elaborate and accurately define the recommendations for healthy lifestyles among kidney transplant recipients who have limited physical activity and poor dietary habits.

## Data Availability

The datasets analyzed during the current study are available from the corresponding author on reasonable request.

## Abbreviations

BMI: Body mass index
DASH: Dietary Approaches to Stop Hypertension
DBP: Diastolic blood pressure
eUC: Enhanced usual care
HEI-2010: Healthy Eating Index 2010
ICC: Intraclass correlation coefficients
KTR: Kidney transplant recipient
FIML: Full information maximum likelihood
PA: Physical activity
QoL: Quality of life
SBP: Systolic blood pressure
SCT: Social cognitive theory
TLC4KTx: Technology-based lifestyle modification intervention group
